# Emergent Differential Organization of Airway Smooth Muscle Cells on Concave and Convex Tubular Surface

**DOI:** 10.3389/fmolb.2021.717771

**Published:** 2021-09-28

**Authors:** Yang Jin, Lei Liu, Peili Yu, Feng Lin, Xiaohao Shi, Jia Guo, Bo Che, Yiyuan Duan, Jingjing Li, Yan Pan, Mingzhi Luo, Linhong Deng

**Affiliations:** ^1^ Key Laboratory for Biorheological Science and Technology of Ministry of Education, Bioengineering College, Chongqing University, Chongqing, China; ^2^ Institute of Biomedical Engineering and Health Sciences, Changzhou University, Changzhou, China; ^3^ Department of Mechanics and Engineering Science, College of Engineering, Peking University, Beijing, China; ^4^ State Key Laboratory of Biotherapy and Cancer Center, West China Hospital, Sichuan University, Chengdu, China

**Keywords:** 3D printing, curvature, airway smooth muscle cells, pattern formation, phenotype

## Abstract

Airway smooth muscle cells (ASMCs) exist in a form of helical winding bundles within the bronchial airway wall. Such tubular tissue provides cells with considerable curvature as a physical constraint, which is widely thought as an important determinant of cell behaviors. However, this process is difficult to mimic in the conventional planar cell culture system. Here, we report a method to develop chips with cell-scale tubular (concave and convex) surfaces from fused deposition modeling 3D printing to explore how ASMCs adapt to the cylindrical curvature for morphogenesis and function. Results showed that ASMCs self-organized into two distinctively different patterns of orientation on the concave and convex surfaces, eventually aligning either invariably perpendicular to the cylinder axis on the concave surface or curvature-dependently angled on the convex surface. Such oriented alignments of the ASMCs were maintained even when the cells were in dynamic movement during migration and spreading along the tubular surfaces. Furthermore, the ASMCs underwent a phenotype transition on the tubular (both concave and convex) surfaces, significantly reducing contractility as compared to ASMCs cultured on a flat surface, which was reflected in the changes of proliferation, migration and gene expression of contractile biomarkers. Taken together, our study revealed a curvature-induced pattern formation and functional modulation of ASMCs *in vitro*, which is not only important to better understanding airway smooth muscle pathophysiology, but may also be useful in the development of new techniques for airway disease diagnosis and therapy such as engineering airway tissues or organoids.

## Introduction

One of the most important challenges in the study of bronchial airway development and pathobiology is to uncover the collective cellular behaviors and associated molecular mechanisms that contribute to particular structural patterning during airway tissue morphogenesis and disease. For example, it is well-known that within the tubular wall of bronchial airways, especially the small ones, airway smooth muscle cells (ASMCs) form spirally winding bundles in the circumferential direction (Ebina et al., 1990; Twellaar et al., 1997). Such a pattern of smooth muscle bundles with varying orientation angle has been thought to play important roles in providing and maintaining airway tubular structure with either appropriate circumferential contractility in health or excessive airway narrowing in diseases such as asthma (Ijpma et al., 2017).

However, it remains undisclosed and thus poorly understood how ASMCs self-organize into such pattern around tubular structure *in vivo*, while much attention has been paid to investigate many factors including chemical and physical cues and cell-cell interactions involved in airway branching morphogenesis (Low and White, 1998; Kim et al., 2015). Nevertheless, it is generally recognized that *in vivo* all cell types including ASMCs usually live within various types of curved space such as folds, gulfs, tunnels and tubes, where the cell behavior is postulated to be highly regulated by the physical cues arising from the complex structural and mechanical microenvironment (Huang and Ingber, 1999; Assoian et al., 2019). It is indeed remarkably demonstrated in stem cell differentiation and tumorigenesis that physical properties such as extracellular matrix stiffness and interfacial geometry play key roles in determining cell fate and guiding tissue development (Park et al., 2011; Chaudhuri et al., 2014; Lee et al., 2016).

In our previous work, we have also shown in two dimensions (2D) that ASMCs cultured on planar curved micropatterns can sense the curvature in their microenvironment and change behaviors in differentiation, orientation, and migration accordingly (Xu et al., 2011). Although these findings suggest that curvature does influence the behaviors of ASMCs, it is obvious that the 2D curved micropatterns fabricated on conventional planar cell culture plate can hardly mimic the three-dimensional (3D) geometry of the tubular airway wall. Thus, 3D tubular surfaces are required for *in vitro* study to capture more realistic features of the cellular behavior of ASMCs on tubular surfaces, but engineering fabrication of 3D tubular surface micropatterns for *in vitro* cell study has proved technically challenging.

Only in recent years, several groups have developed such 3D micropatterns and used them to study cells including ASMCs in three dimensions. They have reported that cells exhibit different behaviors on curved, mainly tubular surfaces as compared to flat ones, such as anisotropic patterned migration (Park et al., 2009; Song et al., 2015; Werner et al., 2017, 2019), constrained cell movement and arrangement (Xi et al., 2017; Costa et al., 2020; Simon-Yarza et al., 2021), orientated cell division (Tang et al., 2018), divergent stem cell fate differentiation to form functional tissues (Pilia et al., 2013; Werner et al., 2017). These findings again highlight the great potential of the geometric surfaces in regulating cell behaviors and how bioengineering tools can be utilized to study tubular tissue formation. But the limitation is that most of these studies only considered cells cultured on the inner or concave side of the tubular surface but few considered them on both convex and concave surfaces, which is reasonable because forming luminal cell layer is the most common phenomenon in tubular tissue development. As regards the airway tissue development, however, the ASMCs eventually locate on the convex rather than the concave side (which is for the epithelial cells) of the airway wall (Ebina et al., 1990). Therefore, it would be more relevant to examine how ASMCs behave on convex tubular surfaces as compared to concave ones.

In the present study, we thus sought to develop a method to fabricate tubular substrate with an either concave or convex surface for cell culture which closely matches the confined curved space of small airways in radius of curvature. We then cultured primary ASMCs on both the concave and convex tubular surface micropatterns, and quantitatively determined the collective cell migration, morphogenesis, and phenotype of these cells. We found that ASMCs behave distinctively differently on the concave and convex surfaces, resulting in a morphology of cells orientated with respect to the axial direction either perpendicularly on the concave surfaces or in a radius-dependent manner on the convex surfaces, as well as corresponding reorganization of the cytoskeletal structure and transition between the contractile and proliferative phenotypes. These results provide at least *in vitro* a new phenomenon of emerging differential organization of ASMCs on the two sides of a same tube, which may help further understand the role of physical regulation in the tissue development of bronchial airways, and the tissue pathogenesis in airway diseases as well as the development of new techniques for airway regeneration.

## Materials and Methods

### Fabrication of Concave and Convex Tubular Surfaces on PDMS Substrate

**FIGURE 1:**
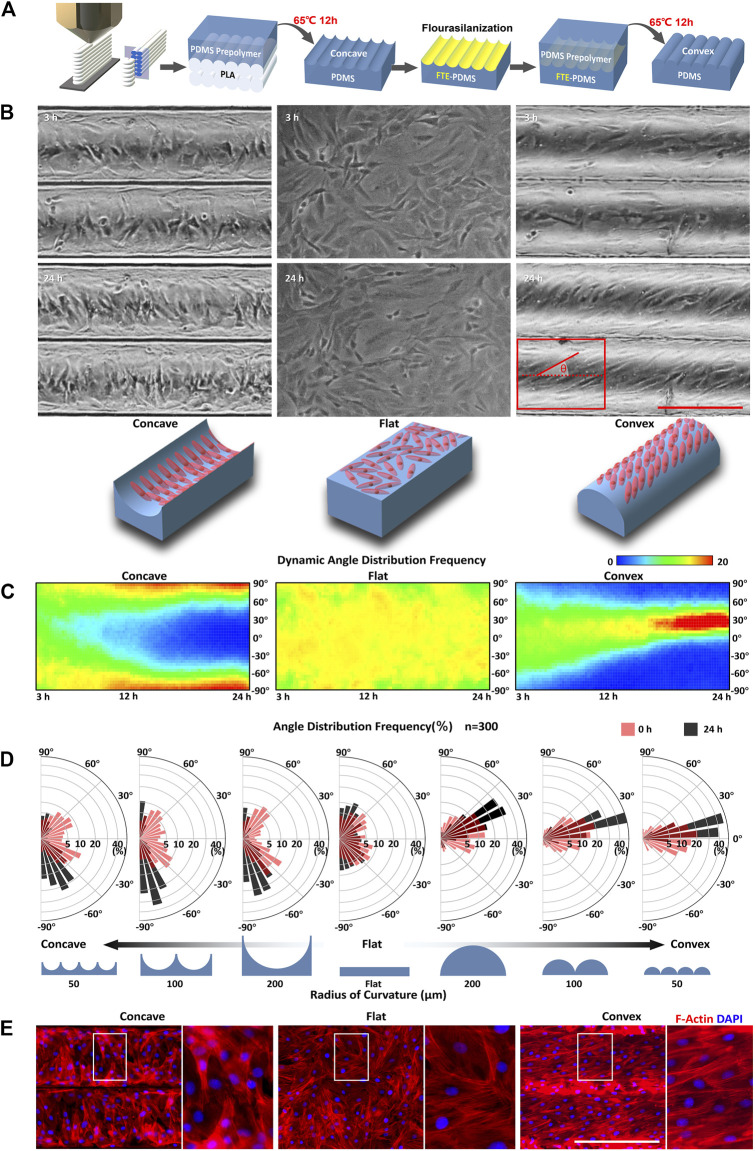
Curvature-driven differential self-organization of ASMCs **(A)** Setup used to fabricate cell culture chips with concave and convex surface substrate **(B)** Representative phase contrast images of ASMCs cultured on a concave surface (*R* = 100 μm; left panel), flat surface (middle panel), and convex surface (*R* = 100 μm; right panel) for 3 and 24 h, respectively, and schematic representation of the observed cell orientation in corresponding concave, flat, and convex surfaces (bottom panel), scale bar = 200 μm **(C)** The thermal diagram shows dynamic orientation of the cells on different curved surfaces from 3 to 24 h, where the color represents the frequency of cells distributed in an angular interval (step size = 5°). The biased angle of cell orientation was defined as the angle between the long (principal) axis of the cell and the cylinder axis as shown in the red diagram in **(B) (D)** Angular histograms showing the quantitative data of biased angle distribution of the ASMCs on concave and convex surfaces with different radii of curvature, and a flat surface for 3 h (red) and 24 h (black). Data of the frequency of biased angle calculated from 300 cells in one experiment that is representative of three independent experiments **(E)** Representative immunostaining images of F-actin (red) and nuclei (blue) for ASMCs on concave (*R* = 100 μm), flat, and convex (*R* = 100 μm) surfaces. Scale bar = 200 μm.

To obtain concave and convex surfaces, a 3D mold with a curved surface was designed and fabricated as shown in Figure 1A. The cuboid mold (length 60 mm, width 0.8 mm, and height 20 mm) designed using SolidWorks 11.0 (SolidWorks Corp., Concord, MA) (Supplementary Figure S1A) was printed via fused deposition modeling (FDM) 3D printer (Makerbot Replicater2, Makerbot, Germany) with polylactide (PLA). The 3D printed PLA molds were actually made of PLA cylinder fiber arrays (Supplementary Figure S1B), and the surfaces of these fibers tended to be cylindrical during 3D printing (Supplementary Figure S2). Therefore, when using different layer heights (0.1, 0.2, 0.4 mm) for printing, the surface of PLA molds presented as cylindrical fiber arrays with different radius of curvature (50, 100, 200 μm).

The concave and convex surfaces were then *trans*-printed successively from these PLA molds (Figure 1A). In order to fabricate a concave surface, PDMS prepolymer (10:1 w/w; Sylgard 184, Dow-Corning) was poured onto PLA molds and cast at 65°C for 12 h. To fabricate a convex surface, the concave surface previously obtained was treated with Trichloro (1, 1, 2, 2H - Perfluorooctyl) Silane (# 78,560–44–8, Aladdin, Shanghai, China) for 30 min, and then PDMS prepolymer was poured onto the concave surface and cast at 65°C for another 12 h again (Figure 1A). Finally, these concave and convex surfaces were successfully fabricated and combined together to make different PDMS chips for different experimental conditions (Supplementary Figure S3 and S4).

All these PDMS chips with concave and convex surfaces were then activated by 0.2 mg/ml sulfo-SANPAH (#22589, Thermo Fisher Scientific, Waltham, MA) using the 365 nm UV LED array for 5 min and rinsed twice with 50 mM HEPES buffered solution (#K301871, Aladdin), and incubated with 50 μg/ml collagen I (# 5,279, Advanced BioMatrix, Inc., Poway, CA) overnight at 4°C for the next cell incubation.

### Cell Culture

Primary ASMCs were isolated from male Sprague-Dawley (SD) rats (6–8 weeks old, purchased from Cavens Lab Animal Co. Ltd., Changzhou, China) following the Guide for the Care and Use of Laboratory Animals published by the Ministry of Health of the People’s Republic of China. Briefly, primary ASMCs were isolated according to the protocol described by Hirst et al. (Hirst, 1996), and then cultured in DMEM-F12 medium (#D0697, Sigma, St. Louis, MO) supplemented with 10% fetal bovine serum (# 10100147, Gibco, Grand Island, NY) and 1% penicillin/streptomycin (# 516,106, Sigma). ASMCs from three to six passage were seeded on each substrate at a density of 1 × 10^5^/cm^2^, and incubated at 37°C with 5% CO_2_. Cells on substrates were observed by phase contrast optical microscopy using a live cell imaging system (AxioVision LE 4.8, Carl Zeiss, Jena, Germany).

### Assessment of Cell Orientation

Quantification of ASMC orientation cultured on the concave and convex surfaces and the cylinder axis was performed using the distribution analysis function in the plugin OrientationJ of ImageJ software (National Institutes of Health, United States) as described by others (Rezakhaniha et al., 2012; Huycke et al., 2019). Briefly, each image would be split into multiple tiny rectangular (50 μm × 50 μm) regions of interest (ROI) that form arrays. In each region, $f_{x}$ and $f_{y}$ are the partial spatial derivatives of the gray-scale map f(x,y) , along directions x and y , respectively. The weighted inner product is defined as,

$$\begin{array}{c} {\langle f_{x},f_{y}\rangle }_{w}=\iint_{R^{2}}w\left(x,y\right)f_{x}\left(x,y\right)f_{y}\left(x,y\right)dxdy \end{array}$$ (1)

Where w(x,y)≥0 is the Gaussian weighting function that specifies the ROI, which is a normalized square window centered (*x*_0_, *y*_0_) on the ROI. Then the directional orientation of cells within each ROI was calculated as follows:

$$\begin{array}{c} \theta =\frac{1}{2}\arctan\left(2\frac{{\langle f_{x},f_{y}\rangle }_{w}}{{\langle f_{y},f_{y}\rangle }_{w}-{\langle f_{x},f_{x}\rangle }_{w}}\right)\; \end{array}$$ (2)

Only the θ values of the ROIs on curved surfaces perpendicular to the perspective were considered available.

### Assessment of Cell Proliferation

The ASMCs were incubated on curved surfaces with different radius of curvature for 48 h. 10 μL of the CCK-8 solution (# CA1210, Solarbio, Beijing, China) was added into the medium on each surface for 4 h incubation, then the cultured medium was transferred to a 96-well plate. The 450 nm absorbance was measured via a microplate reader (# Infinite F50, TECAN, Männedorf, Switzland) to calculate the number of vital cells on each surface.

### Assessment of Cell Migration

To investigate the migration of ASMC monolayer on the concave or convex surface, a wound healing model experiment was performed. Briefly, a soft PDMS strip blocker (0.8 mm width) complementary with the concave or convex surface was placed in the center on the curved surface. Then, ASMCs were plated on the curved surface. After the ASMCs formed a monolayer, the blocker was peeled off and the cells began to migrate towards the cell-free space that was previously concealed by the blocker. The relative cell migration over 24 h was quantified based on the changes in the wound healing area at 0 and 24 h, and the migration rate was defined by the ratio of the wound healing area on each curved surface to that on a flat surface. Cell migration and rearrangement behavior was complete at 24 h and remained unchanged afterwards for up to 72 h. Therefore, only data from 0 to 24 h are presented for all the experiments in this study.

### Immunostaining of ASMCs

For evaluation of the cytoskeletal structure of ASMCs, the cells were fixed with 4% paraformaldehyde (# C104190, Aladdin) for 15 min and then permeabilized with 0.1% Triton X-100 (# T109026, Aladdin) for 15 min at room temperature. Cells were blocked with 5% BSA for another 30 min before incubated with primary mouse monoclonal antibody anti-α-SMA (1:100, # ab7817, Abcam, Cambridge, MA) overnight at 4°C, and then incubated with Alexa Fluor 488-conjugated secondary antibody (1:200, # ab150117, Abcam). Rhodamine phalloidin (1:200, # 51,927, Sigma) was used for labeling stress fibers. DAPI (# D9542, Sigma) was used for visualization of the cell nucleus and then the stained cells were viewed and imaged with a confocal microscope (LSM-700, Carl Zeiss, Jena, Germany).

### RNA Isolation and qRT-PCR Analysis

Total RNA was isolated from cells using an animal RNA isolation kit (# R0026, Beyotime Biotechnology, China). Reverse transcription of RNA was performed using Mir-X miRNA First-Strand Synthesis kit (# 638,315, Takara Bio, Shiga, Tokyo, Japan). Quantitative reverse-transcription PCR (qPCR) was performed and monitored using TaqMan probes (# 450,025, Applied Biosystems, Carlsbad, CA) and primers (listed in Supplementary Table S1). Calibrations and normalizations were done using the 2^-∆∆CT^ method, where ∆∆CT = C_T_ (target gene) - C_T_ (reference gene), while the reference gene was 18S.

### Statistical Analysis

All data were presented as the mean ± standard error of mean (S.E.M.) from experiments that were performed at least with three independent replicates. Statistical analyses were performed with one-way analysis of variance (ANOVA) followed by *t*-test using GraphPad Prism software. A level of *p* < 0.05 was considered statistically significant.

## Results

### ASMCs Self-Organized Into Different Morphological Patterns on Concave and Convex Surfaces

To monitor the self-organization of ASMCs on curved surfaces, primary ASMCs were homogeneously seeded onto concave and convex substrates simulating the topographic features of human small airways, and then the cell arrangement was observed for up to 24 h using time-lapse imaging technique. As shown in Figure 1B, after attaching to the substrate for 3 h, cells randomly distributed and oriented without a specific pattern on the concave (*R* = 100 μm), flat, and convex (*R* = 100 μm) surfaces. However, after attaching for 24 h, the ASMCs on the concave and convex surfaces emerged, unexpectedly, as two distinguishable alignment patterns, aligning in either perpendicular direction to the cylinder axis on a concave surface (left panel) or in a certain angle to the cylinder axis on a convex surface (right panel), respectively. To clarify the dynamic self-organization of ASMCs on concave, flat, and convex surfaces, the orientation of the cells were quantified consecutively from 3 to 24 h in culture and shown in a thermal diagram as in Figure 1C. The results indicate that the ASMCs gradually aligned closer to a biased angle of 95°(−85°) on the concave surface, while closer to a biased angle of 30° on the convex surface, compared to the random orientation on a flat surface. In addition, we found that the orientation angles of ASMCs were modulated by the concave and convex surfaces in a curvature-dependent manner (Figure 1D). Specifically, when the radius of curvature changed from 100 to 50, the biased angle increased from 95° to 115°on the concave surface, and decreased from 30° to 15° on the convex surface. Since the cellular orientation is usually determined by the cytoskeleton especially F-actin, the F-actin filaments of the ASMCs were visualized as shown in Figure 1E. It clearly shows that the F-actin filaments in the ASMCs also appeared nearly perpendicular to the cylinder axis on the concave surface and aligned at an angle on the convex surface, indicating a different reorganization of the F-actin cytoskeleton led to the differential self-organization pattern of ASMCs on the concave and convex surfaces.

Considering the potential effect of boundary guidance on the cellular patterning on closed surfaces (Cao et al., 2010; He et al., 2015; Yu et al., 2021), we quantified the orientation of ASMCs cultured on an either flat rectangular surface or glass tube with the area or curvature similar to that in the above cases (Supplementary Figure S5 and S6). The results show that the self-organization behavior of the ASMCs did not occur on the planar surface with boundary, but occurred on the inner and outer surfaces of the glass tube without boundary, which rules out the impact of boundary guidance on these cell self-organization. Together, our data suggest that curvature alone initiated the self-organization of ASMCs into different patterns on concave and convex surfaces.

### Stress Fibers Helical Arrangement on the Concave and Convex Surfaces

**FIGURE 2:**
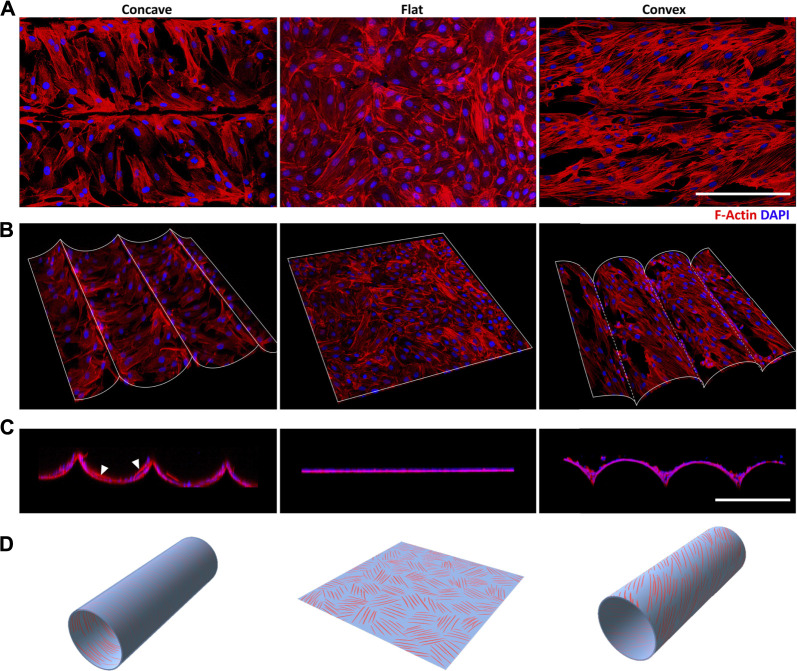
Curvature-induced stress fibers (SFs) helical arrangement of ASMCs **(A)** The distribution of Rhodamine labeled SFs (red) and DAPI labeled nuclei (blue) obtained via the projection of section-scanned image sequences from laser scanning confocal microscopy (*R* = 100 μm) **(B)** Free view and **(C)** side view of 3D reconstructed ASMCs layer on the curved surfaces **(D)** The schematic illustration shows three different patterns of SFs helical distribution induced by concave, flat and convex surfaces. Scale bar = 200 μm.

To further explore the underlying mechanisms of ASMCs alignment on the curved surface, we performed 3D reconstruction of the obtained fluorescence image sequences of the cellular stress fibers (SFs). The results show that the SFs in the ASMCs cultured on curved surfaces exhibited similar behavior of directional alignment as to that of the cells long axis (Figure 1A). Specifically, the SFs also aligned in biased angles and formed helical structures in 3D space with varying angles and pitches of the spirals, as shown in the schematic diagram in Figure 2B,D. Interestingly, the side view of the SFs on the concave surface presented some SFs that were anchored to the surface at two ends but straightened and suspended in the middle like a bridge structure (as pointed at with white arrowheads in Figure 2C), while on the convex surface the SFs appeared to attach closely to the substrate and no bridge-like structures were seen. The helical arrangement of the SFs in ASMCs cultured on a convex surface were indeed similar to that of the airway smooth muscle bundles found *in vivo* (Ebina et al., 1990; Twellaar et al., 1997), which may enable the collective contraction of cells on a tissue scale.

Besides the directional self-organization, the ASMCs on the convex surface exhibited stronger and more ordered SFs as compared to their counterparts on the concave and flat surfaces (Figure 2A). And the SFs in the ASMCs on the convex surface appeared to be more cross-linked than those on the concave surface, suggesting that the ASMCs on the convex surface (outer side of the airway wall) may be self-organized in this way for more efficient force generation and transmission.

### Cell Alignment Maintained During Migration on the Concave and Convex Surfaces

**FIGURE 3:**
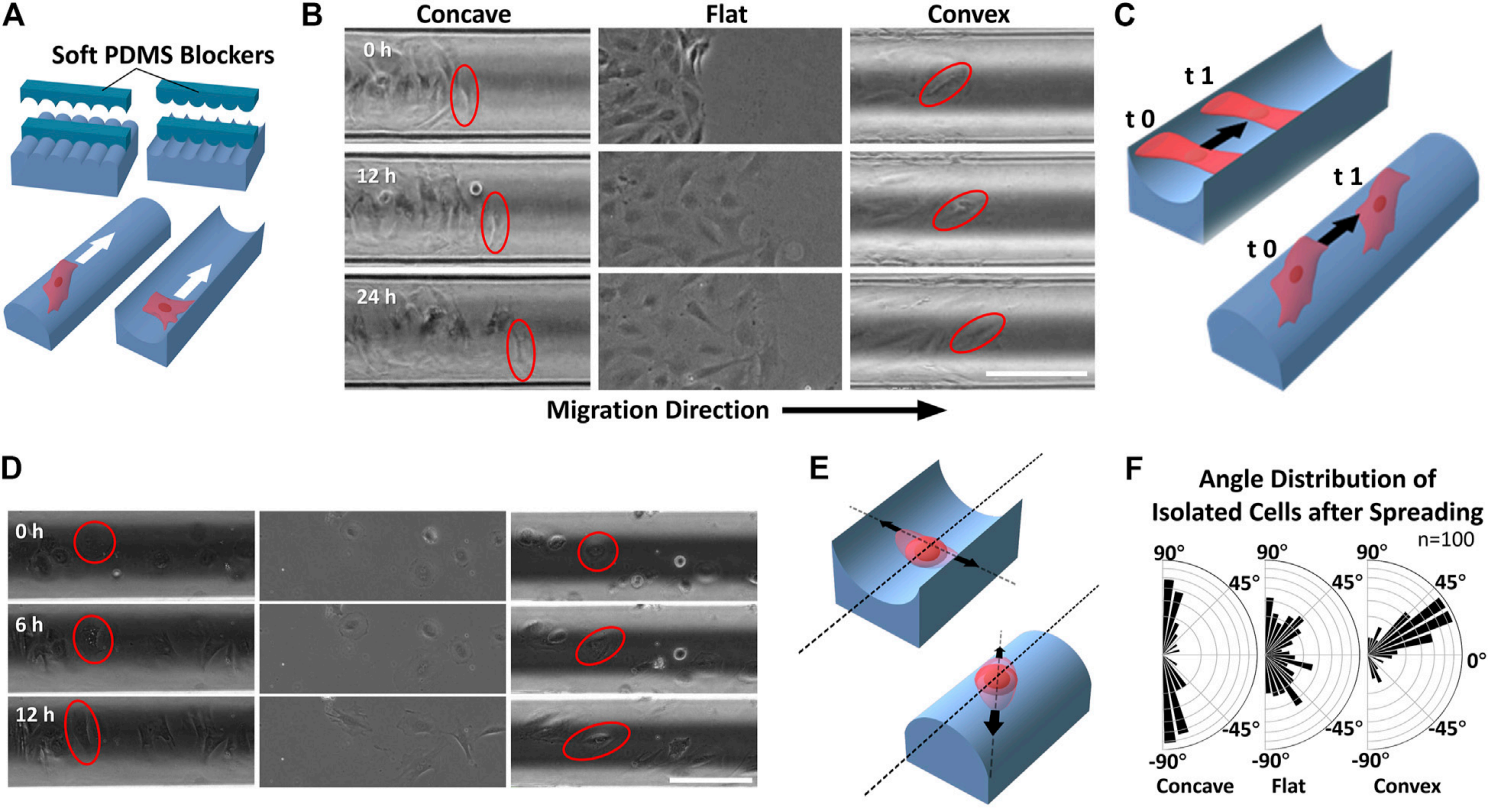
Migration of ASMCs on curved surfaces **(A)** A wound model experiment to provide cell-free gaps with well-defined geometry in the collective cells by placing and removing soft PDMS blockers **(B)** Representative time-lapse images of longitudinal migration (along the cylinder axis) on different surfaces during 24 h. The red oval frames show the alignment of the leading cells during movement **(C)** Schematic illustration of the two migration modes of ASMCs on concave and convex surfaces **(D)** Representative time-lapse images of scattered single ASMCs spreading on different surfaces during 12 h. The red circle/oval frames mark the changing shape of the cell during spreading **(E)** Schematic illustration of the ASMCs spreading along with certain directions on curved surfaces **(F)** Polar plots showing the quantitative data of biased angle distribution of isolated ASMCs after 12 h of spreading on curved surfaces (*R* = 100 μm), and a flat surface (n = 100). Scale bar = 200 μm.

Cell migration always contributes to the maintenance of tissue renewal and homeostasis. To study whether the airway surface curvature affects the dynamic morphology of ASMCs, the migration and adhesion behaviors of ASMCs cultured on curved surfaces were observed over 24 h via wound-healing experiment. As shown in Figure 3A, the ASMCs were first cultured on the concave, flat and convex surfaces while a soft PDMS blocker was placed in the middle of the surface. Once the cells well attached to the surface, the blocker was removed, and the cells began to migrate in a directional way towards the cell-free region that was previously covered by the blocker. Interestingly, even though the cells were in dynamic movement during migration, the leader cells (labeled by the red circles in Figure 3B) on the concave and convex surfaces always maintained their original orientations in alignment (0 vs 12 and 24 h in Figures 3B,C).

In addition, when a scattered single ASMC (shown with red circles in Figure 3D) attached to the curved surface, it spread from a spherical shape to a spindle shape with a certain orientation over 12 h. Consistent with the collectively migrated cells as described above, the scattered single ASMC also self-oriented along the direction either perpendicular or about 30° to the cylinder axis on the concave or convex surface, respectively (Figures 3E,F). These results further illustrate the potent effect of curvature on maintaining the morphological stability of airway smooth muscle during development and wound healing.

### Phenotypic Transformation of ASMCs on the Concave and Convex Surfaces

**FIGURE 4:**
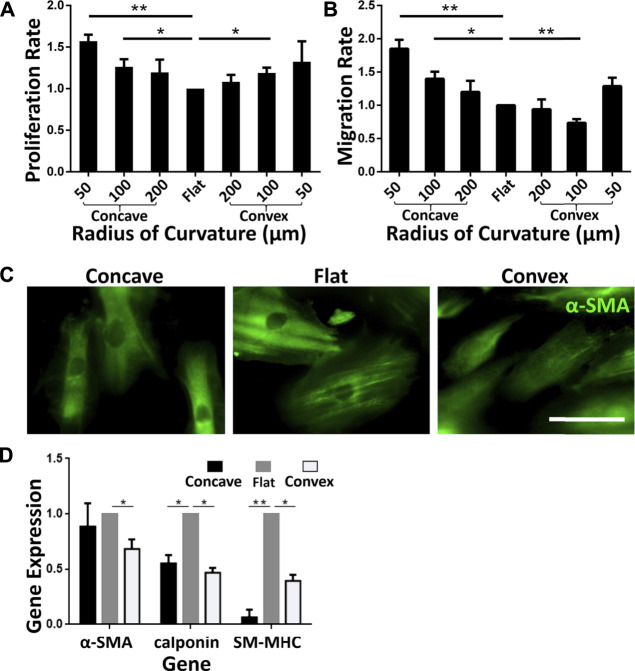
Phenotypic transformation of ASMCs on curved surfaces **(A)** The proliferation rate of cells on different surfaces **(B)** Longitudinal migration rate on different curved surfaces **(C)** Fluorescence profiles of α-SMA of ASMCs on different curved surfaces (concave: *R* = 100 μm, flat, convex: *R* = 100 μm, from left to right), scale bar = 50 μm **(D)** Fold changes of mRNA expression of α-SMA, calponin and SM-MHC in ASMCs cultured on the concave and convex surfaces compared to the flat sruface. Data are presented as mean ± S.E.M, n = 3, *p < 0.05, **p < 0.01.

Physiological ASMCs exist in two different phenotypes, i.e. either proliferative or contractile phenotype which is associated with different pathophysiological processes in airways (Low and White, 1998). It has been revealed that 2D curvature modulates the phenotype of ASMCs, suggesting tubular curvature may also have a similar impact (Xu et al., 2011). Therefore, we analyzed the proliferation, migration, and expression of phenotype-associated biomarkers of ASMCs inoculated on the concave and convex surfaces. The results as shown in Figure 4A indicate that the proliferation rate of ASMCs cultured on the concave and convex surfaces for 24 h was increased in a curvature-dependent manner as compared to the flat surface. And the migration rate of the cells exhibited a similar trend of dependence on the surface curvature (Figure 4B). These findings imply that the ASMCs on the curved and flat surfaces might be of different phenotypes.

To confirm the phenotype of the ASMCs, we fluorescently labeled the cells with alpha-smooth muscle actin (α-SMΑ), an important reference marker for the contractile phenotype of ASMCs. As shown in Figure 4C, the ASMCs on flat surface exhibited strong filamentous structures labeled by α-SMΑ, representing the contractile phenotype of the cells, but the cells on concave surface showed α-SMΑ staining at a reduced level, and on convex surface even a diffusion-like state. Furthermore, we obtained gene expression of representative markers related to ASMCs phenotype (Halayko et al., 1996; Halayko and Solway, 2001) by qPCR after cells cultured on different surfaces for 48 h. As shown in Figure 4D, the results indicate that the expressions of SM-MHC and calponin were down-regulated on both concave and convex surfaces, indicating the reduction of the contractile phenotype of ASMCs. Taken together, the results of proliferation, migration, and phenotype marker expression indicate that curved surfaces induced a phenotypic switching in ASMCs from contraction to proliferation.

## Discussion

In this study, we first revealed that primary ASMCs cultured on a concave (inner) and convex (outer) side of the tubular substrate self-organized into different directional patterns. In general, ASMCs cultured on the concave surface adapted into an aligned arrangement perpendicular to the cylinder axis. However, the ASMCs cultured on the convex surface adapted to a certain angle relative to the cylinder axis, depending on the radius of curvature. Additionally, our data support the role of curvature in mediating dynamical behaviors of ASMCs: the alignment of the cells remained constant in these two kinds of curved surfaces during migration and spreading. Notably, ASMCs cultured on curved surfaces present a proliferation phenotype instead of a contractile phenotype on the flat surface. These findings unveil the self-organization potential of ASMCs to form a differential morphological and functional pattern *in vitro* under a physical microenvironment with different curvatures.

It is difficult for cells to escape the constraints of uneven terrain in the complicated 3D microenvironment. In previous studies, various curvature-induced behaviors have been observed in epithelial cells, fibroblasts, tumor and stem cells (Song et al., 2015; Yevick et al., 2015; Werner et al., 2017; Bade et al., 2018). Thus, it is highly desirable to perform *in vitro* study of ASMCs on tubular surfaces that may simulate the small bronchial airway tubes with corresponding curvature. However, it has been technically challenging to make such models in batches for experiment due to high cost and time-consuming labor. To some extent, our technique based on 3D printing provides an in-house solution to this problem. In our strategy, we realized and utilized the surface of materials printed with PLA filaments via FDM to form a cylinder array. Based on these cylinder array models with different radii, both concave and convex tubular surfaces with different curvatures could be easily made via PDMS molding to simulate the curved environment of tubular structure of early airway tissue.

Therefore, in our experimental set-up, we reproduced the spiral arrangement and phenotypic transformation of ASMCs on the tubular surfaces similar to those occurred in bronchial airways *in vivo* (Choi et al., 2014; Werner et al., 2018). Moreover, we demonstrate that the SFs of the ASMCs also aligned along the principal direction of the cells, which may be required for a strong circumferential tension (Ijpma et al., 2017) and promoting the functionalization of the cell groups. On the other hand, curvature-induced self-assembly of ASMCs may directly affect the arrangement of cells in the embryonic cell-scale airway, thus affecting subsequent airway development (Kim et al., 2015). Furthermore, our data indicated that ASMCs on curved surfaces could maintain their “inherent” direction during migration and spreading, as a positive feedback to control the stable morphology of the airway smooth muscle even if the tissue is constantly self-renewing or damaged.

The biological properties of airway smooth muscle depend largely on the phenotype of the ASMCs, either a contractile or a proliferative type. Our results show that the ASMCs on the concave and convex surfaces are in a less contractile state compared with their counterparts on a flat surface, indicating that the curvature constraint of the tubular tissue might play a role in maintaining the function of ASMCs. And the localized differentiation of smooth muscle during airway development might benefit from these influences of the curved surface on cell fate. It further points out the limitation of the planar cell culture system, for missing the curvature constraints that are necessary for reproducing the physiological functions of the ASMCs. It is also worth noting that the self-organization of ASMCs on the curved surfaces may be useful in optimizing the approach of arranging smooth muscle cells to form bionic tubes, in the development of airway tissue engineering (Choi et al., 2014; Werner et al., 2018). In addition, given the different phenotypes of ASMCs on the flat and curved surface, the necessity is emerged to establish pathological and drug-screening models on the curved surface.

It should also be noted that we used passaged, instead of freshly isolated primary rat ASMCs in this study despite that the latter are more physiological. This is due to the current technical challenge to obtain sufficient amount of highly adherent freshly isolated ASMCs to form high density inoculation with cell patterning. The arrangement pattern of cells we identified are thus only pertinent to the rat ASMCs. Furthermore, in this study, all the substrate models were in semi-tube shape, which is actually a compromise to the full round tube model considering the convenience of model fabrication and experimental observation. These limitations require further studies to be addressed in order to fully reveal the *in vivo* behavior of human ASMCs in the bronchial airways during development and in health and disease.

In conclusion, we first successfully established 3D printing-based chips with cell-scale tubular curvatures. Based on these concave and convex surfaces simulating airway topographic microenvironments, we revealed two emergent self-organization patterns of ASMCs and intracellular SFs on the concave and convex surfaces in a curvature-dependent manner. Moreover, tubular curvatures induce the phenotype switching in ASMCs. These results together suggest that the tubular curvature in airways has an essential role in modulating the morphology and function of ASMCs. These findings provide novel insights for the understanding of lung development, regeneration, and functional maintenance as well as the potential of establishing novel drug screening models for ASM.

## Data Availability

The datasets presented in this study can be found in online repositories. The names of the repository/repositories and accession number(s) can be found in the article/Supplementary Material.
