# Supplementary material for: Emergent Differential Organization of Airway Smooth Muscle Cells on Concave and Convex Tubular Surface
Source: Front Mol Biosci. 2021 Sep 28;8:717771. doi: 10.3389/fmolb.2021.717771 (PMC8505749; doi:10.3389/fmolb.2021.717771)
Supplement: Supplementary file 3 [file Table1.DOCX]

**Supporting information**

**Emergent Differential Organization of Airway Smooth Muscle Cells on Concave and Convex Tubular Surface**

Yang Jin ^1^, Lei Liu ^2^, Peili Yu ^2^, Feng Lin ^3^, Xiaohao Shi ^2^, Jia Guo ^2^, Bo Che ^2^, Yiyuan Duan ^4^, Jingjing Li ^2^, Yan Pan ^2^, Mingzhi Luo ^2*^, Linhong Deng^1,^ ^2*^

^1^ Bioengineering College, Chongqing University, 174 Shapingzheng Street, Shapingba District, Chongqing, 400044, China

^2^ Institute of Biomedical Engineering and Health Sciences, Changzhou University, 1 Gehu Road, Wujin District, Changzhou, Jiangsu 213164, China

^3^ Department of Mechanics and Engineering Science, College of Engineering, Peking University, Beijing 100871, China.

^4^ State Key Laboratory of Biotherapy and Cancer Center, West China Hospital, Sichuan University, Chengdu 610041, P. R. China

^*^Corresponding author:

Mingzhi Luo, E-mail: lmz@cczu.edu.cn

Linhong Deng. E-mail: dlh@cczu.edu.cn.

**
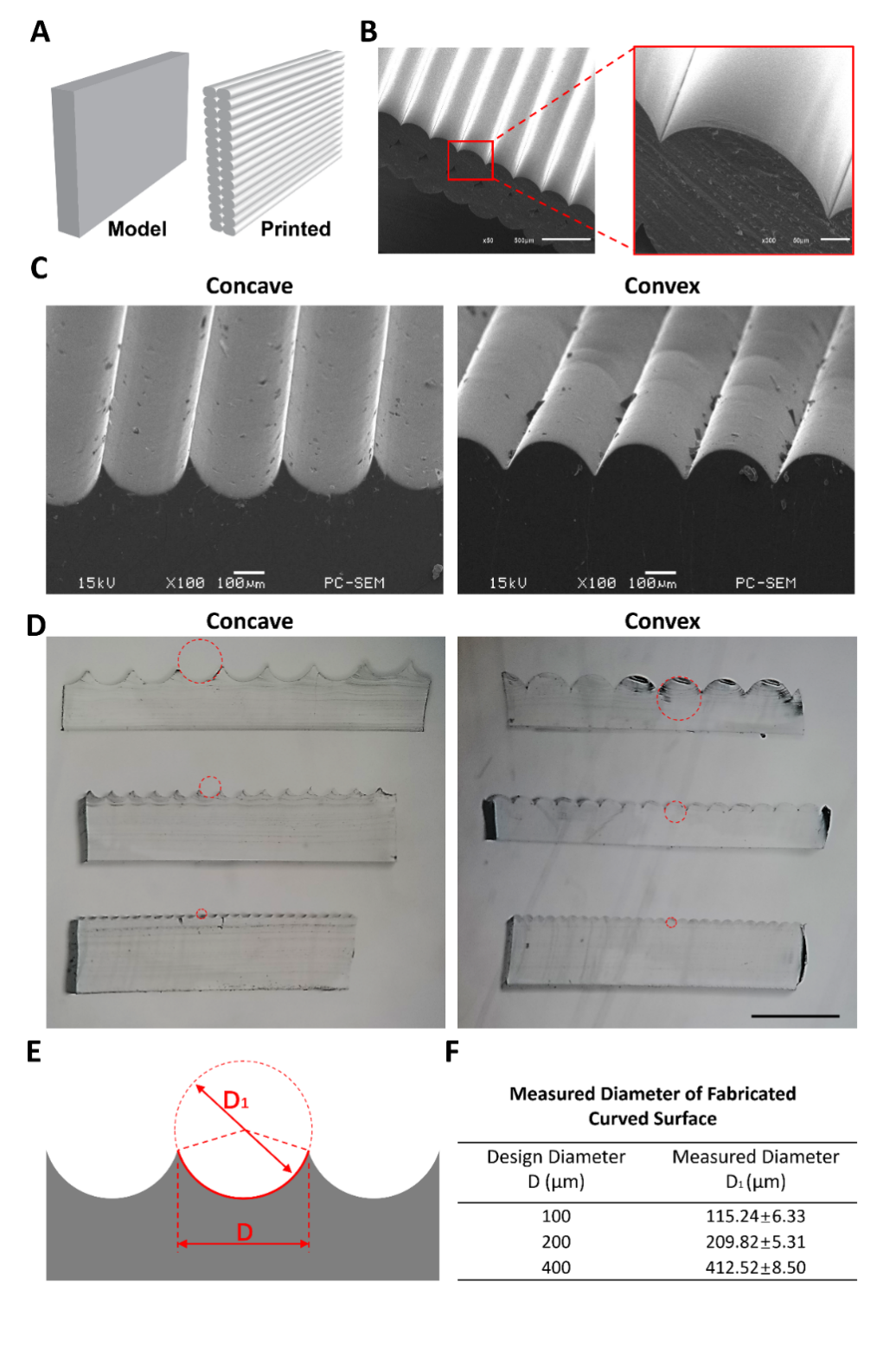
FIGURES**

**Figure S1 Fabrication and calibration of the curved surfaces on PDMS substrates.** (A) Schematic of a thin square designed with SolidWorks (left), and the 3D printed object (right) in detail. (B) The structure of PLA molds and PDMS substrates were observed using a scanning electron microscope (SEM) (SUPRA 55, Zeiss). (C) PDMS substrates with concave and convex surfaces. (D) The PDMS substrates containing the curved surfaces were sliced to measure the actual diameter of the curved surfaces, the red dotted line circle marked the circle to which the curved arc belonged. (E) Indication of the relationship between the designed diameter and actual diameter of curved surfaces, where D is the designed diameter and D1 is the actual diameter. (F) The correspondence between D and D1 is displayed in the table, the data were presented as mean ± S.E.M. Scale bar=800μm.


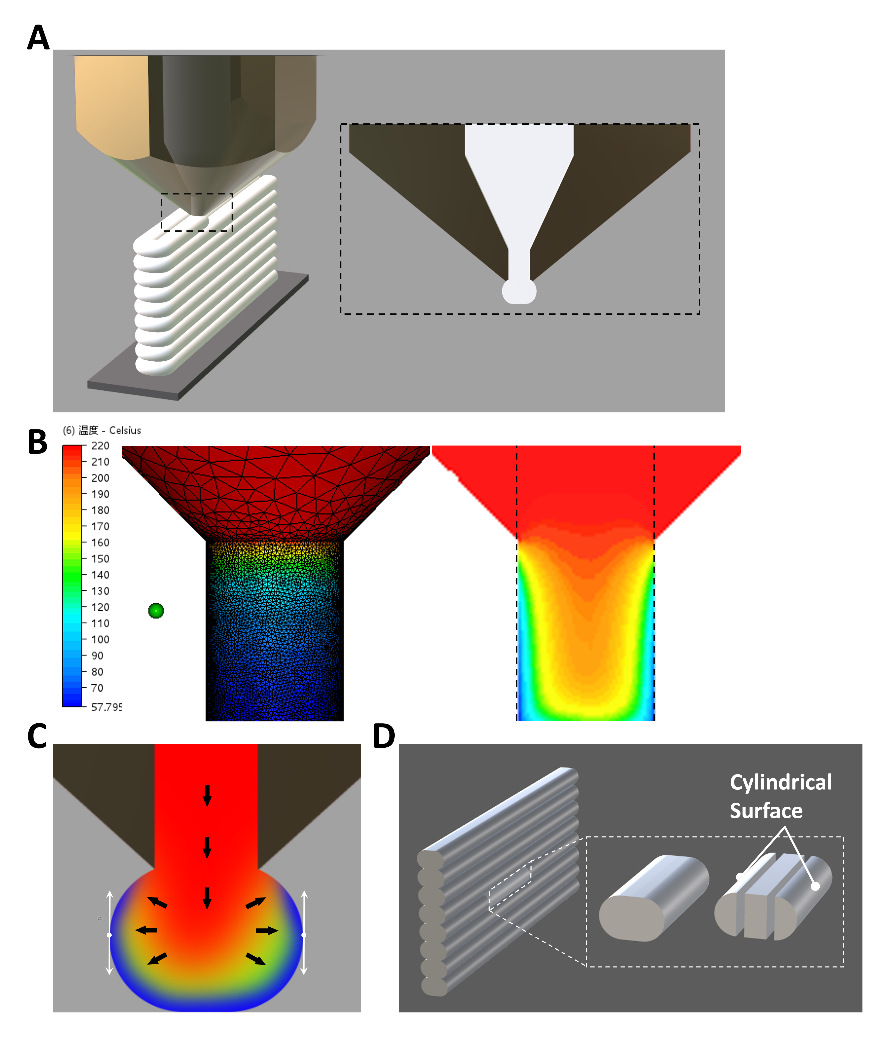
**Figure S2** **The** **thermodynamic simulation of PLA extrusion molding.** To obtain the heat distribution law of PLA at the nozzle outlet during the 3D printing process, thermodynamic simulations were performed using software Autodesk CFD. Boundary conditions were set as follows: shower temperature constant at 225°C, gush out of PLA in static state, the ground was the PLA at room temperature, air flows horizontally at a speed of 20mm/s. (A) Schematic diagram of 3d printing process for FDM and cross section of sprinkler. (B) The thermodynamic simulation image of the heat distribution of PLA in the process of FDM 3D printing. The outer surface of the extruded PLA wire was rapidly cooled at room temperature, while the inner part was still at a high temperature. (C) Schematic illustration of uneven distribution of viscosity varies. The outer surface (shell) cooled by PLA tended to be circular under internal pressure. (D) The surfaces of the 3D-printied fibers were cylindrical.


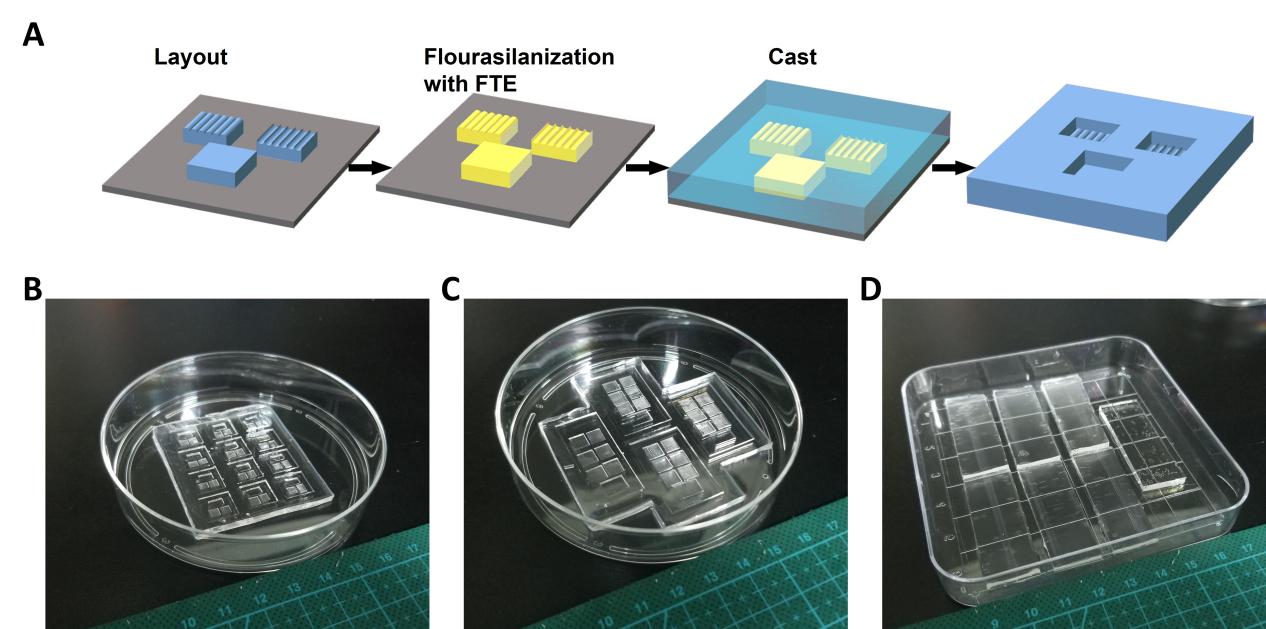


**Figure S3** **Fabrication of different chip** **with curved surfaces.** (A) The fabrication procedure of the chip with compositive curved surfaces, the PDMS substrates with curved surfaces were cut into a specific size and kept a layout on the glass surface, then flourasilanizated with FTE, and finally poured with PDMS to get the final mold. (B) Each unit contained concave surface (R = 100 μm), planes and convex surface (R = 100 μm) for long observation or compositive batch experiments. (C) Each unit contained concave surface (R = 50/100/200 μm), planes and convex surface (R = 50/100/200 μm) for the observation of the cell behavior on curved surfaced with different gradients. (D) The unit had a large area and could be used in experiments that require a large number of cells, such as PCR or WB. Both chips in (B) and (C) were thin for convenient optical observation, chips in (D) were thick to conveniently collect cells.

**Figure S4** **Device for longitudinal migration.** Obstacles shown in (A) could be affixed on the curved surfaces shown in (B) to block the cell migration. Before the cell was inoculated, this step needed to be manually affixed under stereoscopic microscope as shown in the (C), and manually removed after the cell was inoculated.


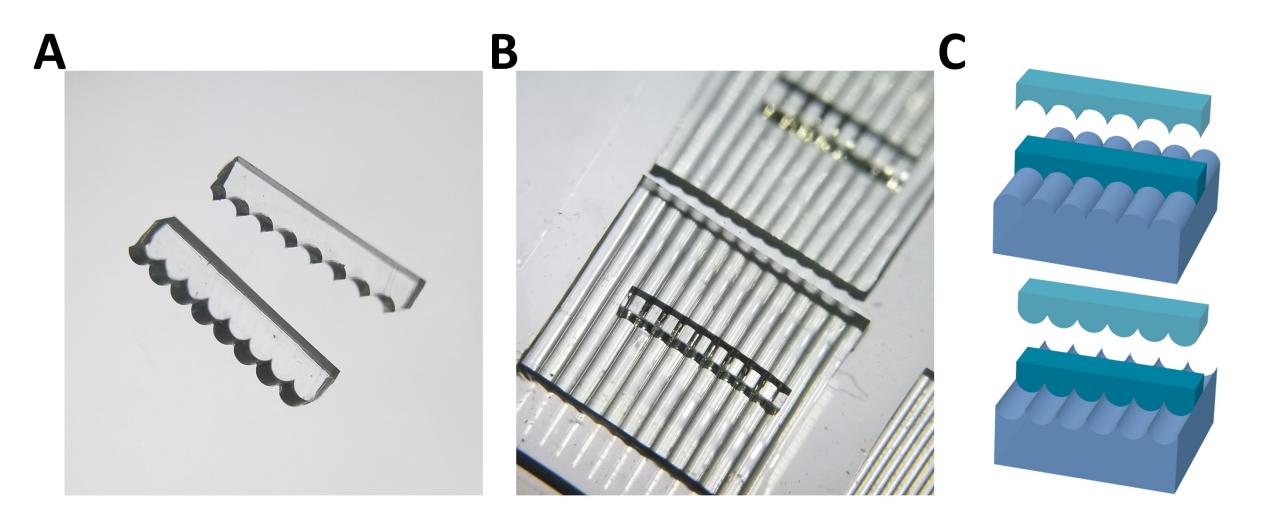

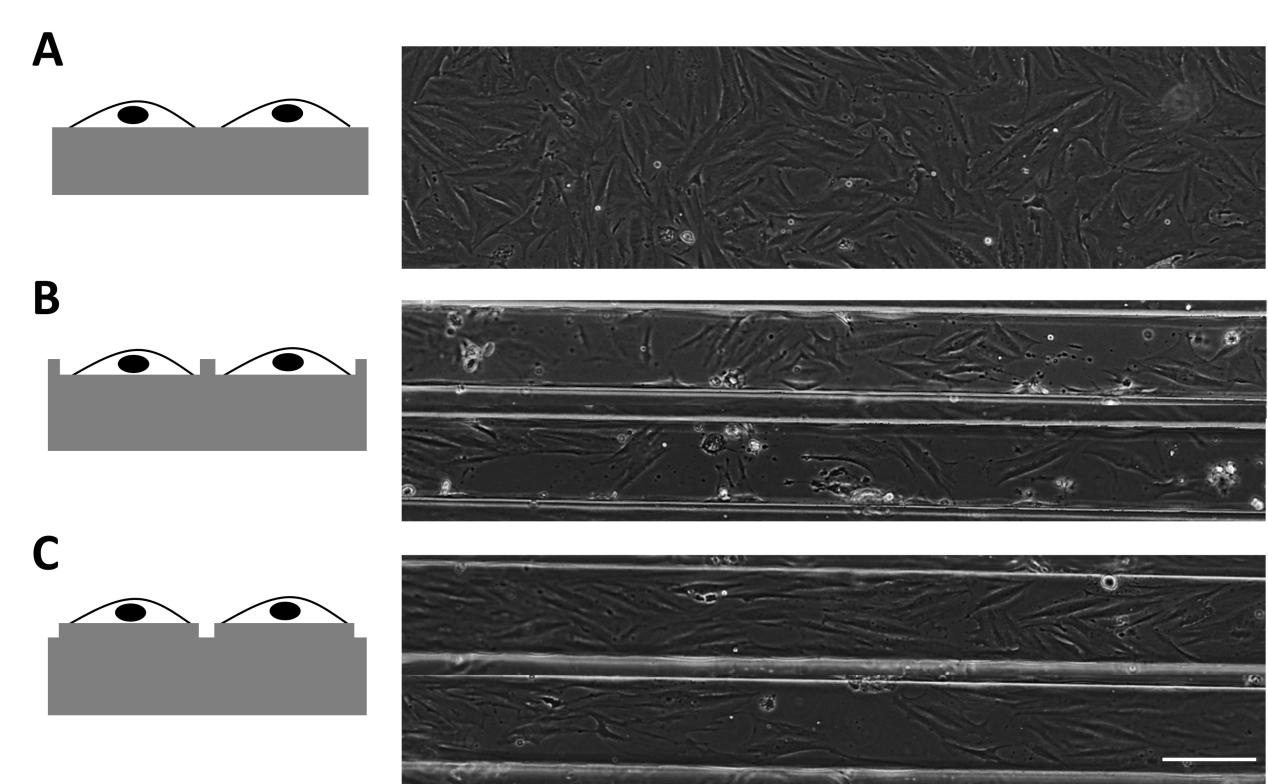


**Figure S5** **The arrangement of cells on a rectangular border stripe.** All the left pictures in (A) (B) (C) showed the cross section of the striped substrates, and the right images showed the arrangement of ASMCs on the plane striped pattern after 24h. (A) was the borderless plane control. (B) was a sunken rectangular groove which was used to simulate the effect of the boundary of the concave substrate on the arrangement of cells. (C) was a bulging rectangular platform used to simulate the effect of the boundary on the convex surface on the arrangement of cells. Scale bar=100μm.


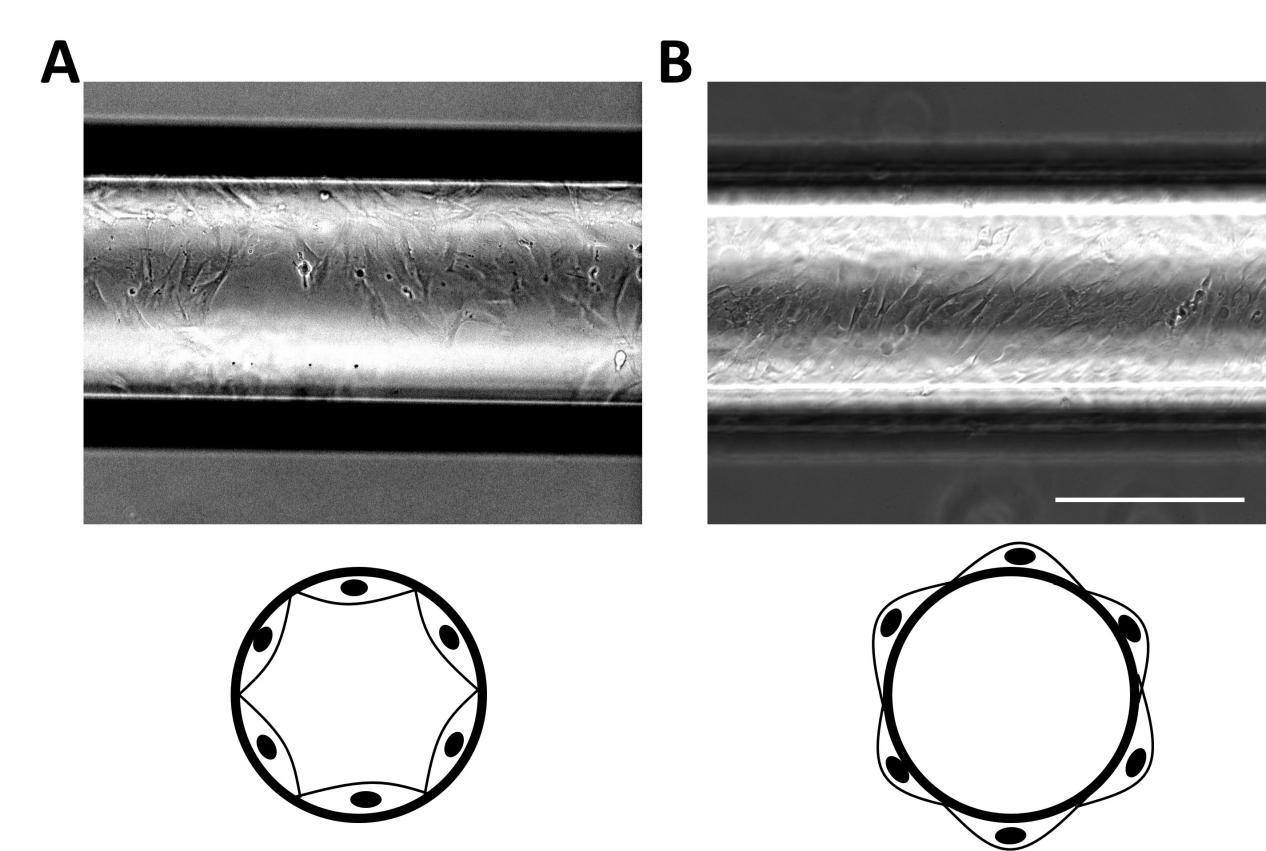


**Figure S6** **Pattern formation of ASMCs on the inner and outer surface of the tube.** The phase contrast images showed the behavior of ASMCs on the borderless tubular surface after inoculation 24h. (A) and (B) showed the inner and outer surface of the tube respectively, these two arrangement patterns were similar to those of ASMCs on the substrates. Scale bar=200μm.

**SUPPLEMENTARY TABLE**

**Supplementary Table 1.** List of primers used in qRT-PCR.

| **Gene** | **Forward** | **Reverse** |
| --- | --- | --- |
| **α-SMA** | CATCCGACCTTGCTAACG | CCAGAGTCCAGCACAATAC |
| **calponin** | TTCCGCACACTTTAACCGAGGTCG | TGGTGCCAGTTCTGGGTTGACT |
| **SM-MHC** | CGCAACACCACGCCTAAC | GCGGATGCCTTCCAACAC |
| **18S** | CCTTCGCTATCACTGCCATTA | GCTATACTTCCCATCCTTCACG |
