# Supplementary figures and images for: Emergent Differential Organization of Airway Smooth Muscle Cells on Concave and Convex Tubular Surface
Source: Front Mol Biosci. 2021 Sep 28;8:717771. doi: 10.3389/fmolb.2021.717771 (PMC8505749; doi:10.3389/fmolb.2021.717771)

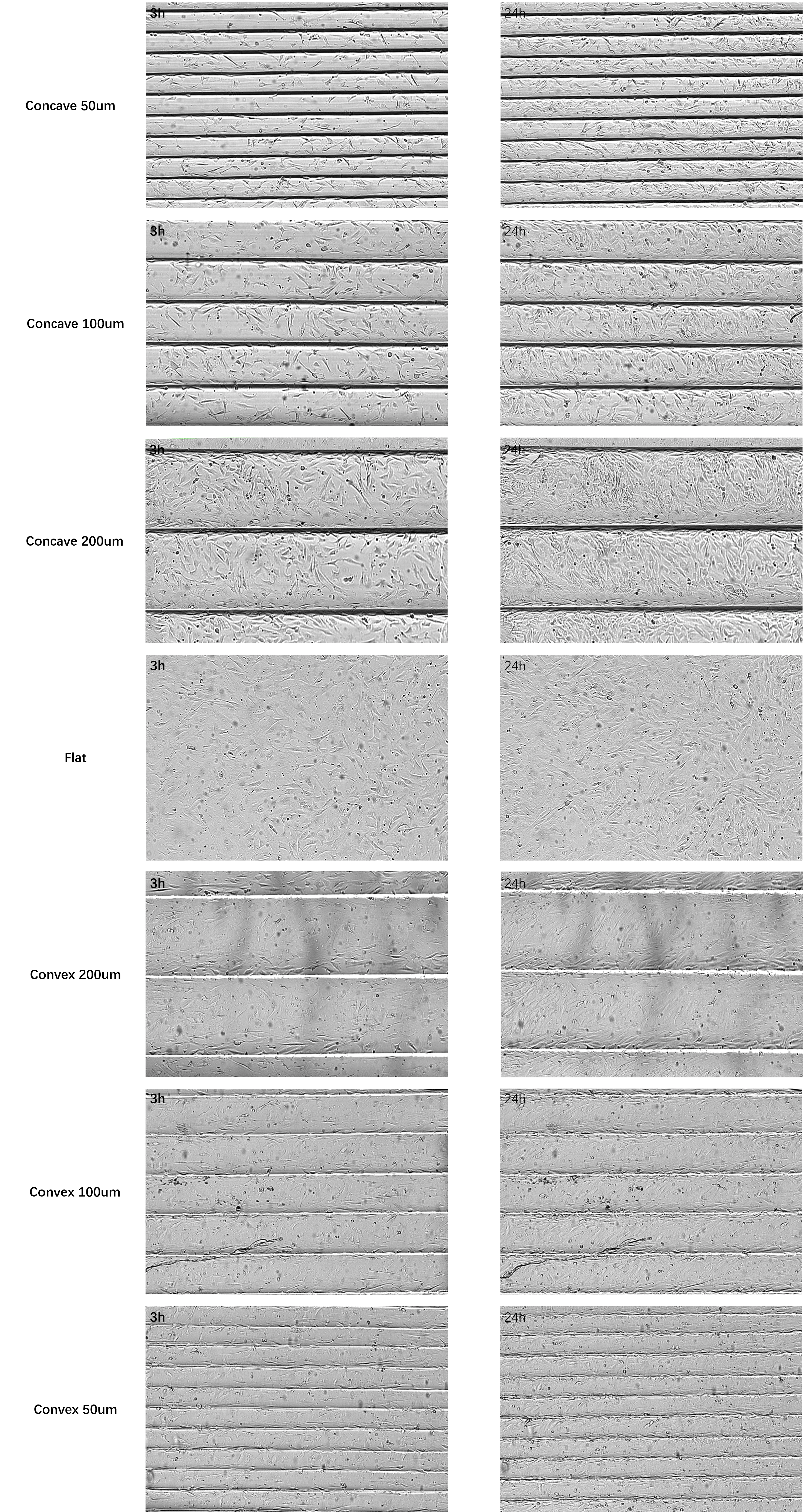

Supplement: Supplementary file 5 [file Image1.PNG]
